# ASPHER Statement: Facing the Fourth Winter of the COVID-19 Pandemic

**DOI:** 10.3389/phrs.2022.1605395

**Published:** 2022-10-03

**Authors:** Rok Hrzic, Nadav Davidovitch, Henrique Barros, Henrique Lopes, Jose M. Martin Moreno, Amanda J. Mason-Jones, Alison McCallum, John Reid, Ralf Reintjes, Mohamud Sheek-Hussein, Judit Simon, Brian Li Han Wong, Lore Leighton, Robert Otok, John Middleton

**Affiliations:** ^1^ Department of International Health, Care and Public Health Research Institute – CAPHRI, Maastricht University, Maastricht, Netherlands; ^2^ School of Public Health, Ben Gurion University of the Negev, Beersheba, Israel; ^3^ Institute of Public Health, University of Porto, Porto, Portugal; ^4^ Unit of Public Health, Institute of Health Sciences, Catolica University, Lisbon, Portugal; ^5^ Department of Preventive Medicine and Public Health, Medical School and INCLIVA, University of Valencia, Valencia, Spain; ^6^ Department of Health Sciences, University of York, York, United Kingdom; ^7^ Centre for Population Health Sciences, Usher Institute, University of Edinburgh, Edinburgh, Scotland; ^8^ Department of Public Health and Wellbeing, University of Chester, Chester, United Kingdom; ^9^ Department of Public Health, Hamburg University of Applied Sciences, Hamburg, Germany; ^10^ Institute of Public Health — College of Medicine and Health Sciences, United Arab Emirates University, Al Ain, United Arab Emirates; ^11^ Department of Health Economics, Center for Public Health, Medical University of Vienna, Vienna, Austria; ^12^ The International Digital Health and AI Research Collaborative (I-DAIR), Geneva, Switzerland; ^13^ Association of Schools of Public Health in the European Region (ASPHER), Brussels, Belgium

**Keywords:** COVID-19 pandemic, winter planning, equity, vaccination policy, evidence informed policy, trust, enhanced surveillance

## The Fourth Winter of the Pandemic—What Have We Learned?

The coming winter is the fourth since the COVID-19 pandemic started in late 2019. The rapidity with which novel variants of the virus are emerging and eroding the effectiveness of established tools for the detection, prevention, and treatment of the illness poses an ongoing challenge to healthcare systems, on top of emerging threats such as the MPVX and polio as well as preparations for seasonal influenza, all within the context of pandemic fatigue both for the general public and health workforce [1]. At the same time, policymakers worldwide now possess the experience necessary to stage effective and equitable responses to the pandemic. In this statement, we reflect on the key lessons for managing the ongoing COVID-19 pandemic in the context of multiple intersecting global threats [2].

## The Pandemic Response Should Prioritize Equity

The first lesson is that this is an inequitable pandemic. Most outcomes relevant to COVID-19 follow a social gradient, ranging from contracting the disease [3], experiencing a severe course of the disease [4] and death [5], vaccine hesitancy [6] and not having received a full course of vaccination [7], or experiencing economic hardship [8] and poorer mental health as a result of the pandemic [9]. Any response, therefore, necessitates a strong consideration of equity effects and entails a combination of tailored information dissemination, vaccination campaigns targeted at different levels of the social hierarchy, providing adequate ventilation, encouraging wearing masks in public places like on public transport [10], ensuring that frequent testing and quarantining are financially accessible for all, and welfare policies ameliorating social and economic deprivation.

## Vaccines are Necessary But Not Sufficient

The availability of COVID-19 vaccines has been a turning point in the pandemic and has resulted in fewer new infections, hospital admissions, and severe courses of disease [11]. However, vaccination alone is not sufficient to prepare for the months ahead. Large sections of the population—often those at the bottom of the social hierarchy—will likely remain unvaccinated in light of their vaccine hesitancy and distrust of the government [6, 7, 12]. Globally, the distribution of COVID-19 vaccines remains unequal as wealthy nations stockpile the vaccine at the expense of easy access in lower- and middle-income countries; this not only hinders the effective response in those countries but also perpetuates the worldwide risk of emergent variants [13–17]. In absence of other preventive strategies, the rapid emergence of novel variants in combination with waning immunity after vaccination [18], necessitates the constant development and rollout of booster doses in the absence of other preventive strategies, which is not sustainable over the long term.

## Transparent Evidence-Informed Decision-Making Engenders Trust

An effective response to the pandemic fundamentally requires a relationship of trust and cooperation between governments and their citizens. This relationship is greatly enhanced if key stakeholders and the public are engaged in a fair process for decision-making involving publicity, relevance, revisability, and enforcement [10]. Given the evidence available on the likely effects of various non-pharmacological interventions on the course of the pandemic [19–21] and the availability of reflections on how best to incorporate scientific evidence in political decision-making during the pandemic [22–24], this moment presents an opportunity for governments to restore much-needed confidence in their ability to handle the pandemic and act decisively to protect the public’s health.

## Effective Surveillance is the Key to an Effective Response

Throughout the pandemic, COVID-19 surveillance systems played a key role in shaping effective responses to the pandemic [25]. Nevertheless, differences between countries, regions, and over time in how COVID-19 cases are defined, tested for, and reported have made the consistent and reliable tracking of the COVID-19 burden challenging [26–28]. To support an effective public health response going into the winter, countries will need to do more and more varied surveillance. Various techniques and tactics are available to provide insight into the prevalence of COVID-19 in a population [29], including wastewater surveillance [30]. There is also an ongoing need for genomic surveillance to detect emerging viral variants [31], as well as for monitoring for COVID-19 in healthcare and other occupational settings [32], inequalities in COVID-19 burden between ethnic groups [33], and the prevalence of long COVID [34]. The ECDC and WHO Europe suggest a good standard for surveillance of respiratory viruses—including COVID-19—consists of representative sentinel surveillance systems in primary and secondary care, the targeted surveillance of vulnerable groups, gathering data on illness severity such as hospitalisations, admissions to ICU, and mortality, and genomic monitoring; the data gathered should be sufficiently disaggregated to accurately follow virus- and variant-specific disease incidence by severity, age, and place [35]. Investing in sustainable surveillance systems will not only help us with COVID-19 but also enable effective public health responses to other emerging health threats.

## The COVID-19 Pandemic Will Not be Fought in Isolation

An effective response to COVID-19 this winter will be critical to preserve the capacities of healthcare and public health institutions as they fend off other threats. Overlapping with the COVID -19 pandemic is the global circulation of MPVX [36], and experts warn of a resurgent seasonal influenza after 2 years suppression by public health measures and travel restrictions [37]. The context is also made more challenging by rising geopolitical uncertainty, high inflation driving up costs of living, extreme weather conditions including heatwaves and floods, and other threats [2]. COVID-19 pandemic is still here, yet we learned many lessons. We must take a balanced, sustainable, and holistic perspective to guide us in our interventions [2]. This requires collaboration and solidarity, both within and between countries.

## Author’s Note

The Association of Schools of Public Health in the European Region (ASPHER) is the key independent European organization dedicated to improving and protecting public health by strengthening education and training of public health professionals for practice and research. www.aspher.org.

## Editorial Note

ASPHER is responsible and liable for all contents of ASPHER statements. ASPHER statements are approved by an Editor-in-Chief but not externally peer reviewed.

## References

[1] Fernandes Q Inchakalody VP Merhi M Mestiri S Taib N Moustafa Abo El-Ella D Emerging COVID-19 Variants and Their Impact on SARS-CoV-2 Diagnosis, Therapeutics and Vaccines. Ann Med (2022) 54(1):524–40. 10.1080/07853890.2022.2031274 35132910 PMC8843115

[2] Middleton J Davidovitch N Barros H Lopes H Martin-Moreno JM Mason-Jones AJ ASPHER Statement: Planning for Winter 2022-23. Public health Rev. (2022) 43:1605394. 10.3389/phrs.2022.1605394 PMC9578316 36267592

[3] Baena-Díez JM Barroso M Cordeiro-Coelho SI Díaz JL Grau M. Impact of COVID-19 Outbreak by Income: Hitting Hardest the Most Deprived. J Public Health (2020) 42(4):698–703. 10.1093/pubmed/fdaa136 PMC7454748 32776102

[4] Gustafsson PE San Sebastian M Fonseca-Rodriguez O Fors Connolly AM. Inequitable Impact of Infection: Social Gradients in Severe COVID-19 Outcomes Among All Confirmed SARS-CoV-2 Cases during the First Pandemic Wave in Sweden. J Epidemiol Community Health (2022) 76(3):261–7. 10.1136/jech-2021-216778 34526373 PMC8449839

[5] Woodward M Peters SAE Harris K. Social Deprivation as a Risk Factor for COVID-19 Mortality Among Women and Men in the UK Biobank: Nature of Risk and Context Suggests that Social Interventions Are Essential to Mitigate the Effects of Future Pandemics. J Epidemiol Community Health (2021) 75(11):1050–5. 10.1136/jech-2020-215810 33906905 PMC8098299

[6] Bajos N Spire A Silberzan L EPICOV study group. The Social Specificities of Hostility toward Vaccination against Covid-19 in France. PLoS ONE (2022) 17(1):e0262192. 10.1371/journal.pone.0262192 34990482 PMC8735622

[7] Saban M Myers V Ben-Shetrit S Wilf-Miron R. Socioeconomic Gradient in COVID-19 Vaccination: Evidence from Israel. Int J Equity Health (2021) 20(1):242. 10.1186/s12939-021-01566-4 34749718 PMC8574141

[8] Witteveen D. Sociodemographic Inequality in Exposure to COVID-19-Induced Economic Hardship in the United Kingdom. Res Soc Stratif Mobil (2020) 69:100551. 10.1016/j.rssm.2020.100551 32921869 PMC7474909

[9] Witteveen D Velthorst E. Economic Hardship and Mental Health Complaints during COVID-19. Proc Natl Acad Sci U S A (2020) 117(44):27277–84. 10.1073/pnas.2009609117 33046648 PMC7959574

[10] Hrzic R Peixoto VR Mason-Jones AJ McCallum A ASPHER COVID-19 Task Force. Is it Really Time to Ditch the Mask? BMJ (2022) 377:o1186. 10.1136/bmj.o1186 35545285

[11] Català M Li X Prats C Prieto-Alhambra D. The Impact of Prioritisation and Dosing Intervals on the Effects of COVID-19 Vaccination in Europe: an Agent-Based Cohort Model. Sci Rep (2021) 11(1):18812. 10.1038/s41598-021-98216-0 34552139 PMC8458447

[12] Nafilyan V Dolby T Razieh C Gaughan CH Morgan J Ayoubkhani D Sociodemographic Inequality in COVID-19 Vaccination Coverage Among Elderly Adults in England: a National Linked Data Study. BMJ Open (2021) 11(7):e053402. 10.1136/bmjopen-2021-053402 PMC8313303 34301672

[13] Binagwaho A Mathewos K Davis S. Time for the Ethical Management of COVID-19 Vaccines. Lancet Glob Health (2021) 9(8):e1169–e1171. 10.1016/S2214-109X(21)00180-7 33961810 PMC8096324

[14] Wagner CE Saad-Roy CM Morris SE Baker RE Mina MJ Farrar J Vaccine Nationalism and the Dynamics and Control of SARS-CoV-2. Science (2021) 373(6562):eabj7364. 10.1126/science.abj7364 34404735 PMC9835930

[15] Wagner CE Saad-Roy CM Grenfell BT. Modelling Vaccination Strategies for COVID-19. Nat Rev Immunol (2022) 22(3):139–41. 10.1038/s41577-022-00687-3 35145245 PMC8830981

[16] Wouters OJ Shadlen KC Salcher-Konrad M Pollard AJ Larson HJ Teerawattananon Y Challenges in Ensuring Global Access to COVID-19 Vaccines: Production, Affordability, Allocation, and Deployment. Lancet (2021) 397(10278):1023–34. 10.1016/S0140-6736(21)00306-8 33587887 PMC7906643

[17] World Federation of Public Health Associations. Statement on COVID-19 Immunization and Equitable Access to Vaccines (2021). Available at: https://www.wfpha.org/statement-on-covid-19/ (Accessed September 23, 2022).

[18] Goldberg Y Mandel M Bar-On YM Bodenheimer O Freedman L Haas EJ Waning Immunity after the BNT162b2 Vaccine in Israel. N Engl J Med (2021) 385(24):e85. 10.1056/NEJMoa2114228 34706170 PMC8609604

[19] Flaxman S Mishra S Gandy A Unwin HJT Mellan TA Coupland H Estimating the Effects of Non-pharmaceutical Interventions on COVID-19 in Europe. Nature (2020) 584(7820):257–61. 10.1038/s41586-020-2405-7 32512579

[20] Haug N Geyrhofer L Londei A Dervic E Desvars-Larrive A Loreto V Ranking the Effectiveness of Worldwide COVID-19 Government Interventions. Nat Hum Behav (2020) 4(12):1303–12. 10.1038/s41562-020-01009-0 33199859

[21] Sharma M Mindermann S Rogers-Smith C Leech G Snodin B Ahuja J Understanding the Effectiveness of Government Interventions against the Resurgence of COVID-19 in Europe. Nat Commun (2021) 12(1):5820. 10.1038/s41467-021-26013-4 34611158 PMC8492703

[22] de Campos-Rudinsky TC Undurraga E. Public Health Decisions in the COVID-19 Pandemic Require More Than ‘follow the Science. J Med Ethics (2021) 2020:107134. 10.1136/medethics-2020-107134 33632729

[23] Hodges R Caperchione E van Helden J Reichard C Sorrentino D. The Role of Scientific Expertise in COVID-19 Policy-Making: Evidence from Four European Countries. Public Organiz Rev (2022) 22(2):249–67. 10.1007/s11115-022-00614-z

[24] Vickery J Atkinson P Lin L Rubin O Upshur R Yeoh EK Challenges to Evidence-Informed Decision-Making in the Context of Pandemics: Qualitative Study of COVID-19 Policy Advisor Perspectives. BMJ Glob Health (2022) 7(4):e008268. 10.1136/bmjgh-2021-008268 PMC9023846 35450862

[25] Baker MG Wilson N Anglemyer A. Successful Elimination of Covid-19 Transmission in New Zealand. N Engl J Med (2020) 383:e56. 10.1056/NEJMc2025203 32767891 PMC7449141

[26] Alwan NA. Surveillance Is Underestimating the burden of the COVID-19 Pandemic. Lancet (2020) 396(10252):e24. 10.1016/S0140-6736(20)31823-7 32861312 PMC7836224

[27] Rocco P Rich JAJ Klasa K Dubin KA Béland D. Who Counts where? COVID-19 Surveillance in Federal Countries. J Health Polit Pol L (2021) 46(6):959–87. 10.1215/03616878-9349114 34075406

[28] Karanikolos M McKee M. How Comparable Is COVID-19 Mortality across Countries? Eurohealth (2020) 26(2):45–50.

[29] Mina MJ Andersen KG. COVID-19 Testing: One Size Does Not Fit All. Science (2021) 371(6525):126–7. 10.1126/science.abe9187 33414210

[30] Shah S Gwee SXW Ng JQX Lau N Koh J Pang J. Wastewater Surveillance to Infer COVID-19 Transmission: A Systematic Review. Sci Total Environ (2022) 804:150060. 10.1016/j.scitotenv.2021.150060 34798721 PMC8423771

[31] Robishaw JD Alter SM Solano JJ Shih RD DeMets DL Maki DG Genomic Surveillance to Combat COVID-19: Challenges and Opportunities. Lancet Microbe (2021) 2(9):e481–e484. 10.1016/S2666-5247(21)00121-X 34337584 PMC8315763

[32] Wee LE Sim XYJ Conceicao EP Aung MK Goh JQ Yeo DWT Containment of COVID-19 Cases Among Healthcare Workers: The Role of Surveillance, Early Detection, and Outbreak Management. Infect Control Hosp Epidemiol (2020) 41(7):765–71. 10.1017/ice.2020.219 32391746 PMC7248595

[33] Harawa NT Amani B Abotsi-Kowu C Nwankwo E Ford CL. Using COVID-19 Surveillance Systems to Identify and Monitor Disparities: Best Practices and Recommendations. Ethn Dis (2022) 32(2):151–64. 10.18865/ed.32.2.151 35497401 PMC9037655

[34] Ward H Flower B Garcia PJ Ong SWX Altmann DM Delaney B Global Surveillance, Research, and Collaboration Needed to Improve Understanding and Management of Long COVID. Lancet (2021) 398(10316):2057–9. 10.1016/S0140-6736(21)02444-2 34774190 PMC8580495

[35] World Health Organization Regional Office for Europe, European Centre for Disease Prevention and Control. Operational Considerations for Respiratory Virus Surveillance in Europe (2022). Available at: https://www.ecdc.europa.eu/sites/default/files/documents/Operational-considerations-respiratory-virus-surveillance-euro-2022.pdf (Accessed September 23, 2022).

[36] Martín-Delgado MC Martín Sánchez FJ Martínez-Sellés M Molero Garcia JM Moreno Guillen S Rodriguez-Artalejo FJ Monkeypox in Humans: a New Outbreak. Rev Esp Quimioter (2022) 2022:martin06jul2022. 10.37201/req/059.2022 PMC9728594 35785957

[37] Dhanasekaran V Sullivan S Edwards KM Xie R Khvorov A Valkenburg SA Human Seasonal Influenza under COVID-19 and the Potential Consequences of Influenza Lineage Elimination. Nat Commun (2022) 13(1):1721. 10.1038/s41467-022-29402-5 35361789 PMC8971476

